# Quantifying dominant bacterial genera detected in metagenomic data from fish eggs and larvae using genus‐specific primers

**DOI:** 10.1002/mbo3.1274

**Published:** 2022-04-30

**Authors:** Babak Najafpour, Patricia I. S. Pinto, A. V. M. Canario, Deborah M. Power

**Affiliations:** ^1^ Centro de Ciências do Mar (CCMAR/CIMAR) Universidade do Algarve Faro Portugal; ^2^ International Center for Marine Studies Shanghai Ocean University Shanghai China

**Keywords:** 16S rRNA gene, aquaculture site, abundant microbiota, eggs, genus‐specific primers, larvae, qPCR

## Abstract

The goal of this study was to design genus‐specific primers for rapid evaluation of the most abundant bacterial genera identified using amplicon‐based sequencing of the 16S rRNA gene in fish‐related samples and surrounding water. Efficient genus‐specific primers were designed for 11 bacterial genera including *Alkalimarinus, Colwellia, Enterovibrio, Marinomonas, Massilia, Oleispira, Phaeobacter, Photobacterium, Polarbacerium, Pseudomonas*, and *Psychrobium*. The specificity of the primers was confirmed by the phylogeny of the sequenced polymerase chain reaction (PCR) amplicons that indicated primers were genus‐specific except in the case of *Colwellia* and *Phaeobacter*. Copy number of the 16S rRNA gene obtained by quantitative PCR using genus‐specific primers and the relative abundance obtained by 16S rRNA gene sequencing using universal primers were well correlated for the five analyzed abundant bacterial genera. Low correlations between quantitative PCR and 16S rRNA gene sequencing for *Pseudomonas* were explained by the higher coverage of known *Pseudomonas* species by the designed genus‐specific primers than the universal primers used in 16S rRNA gene sequencing. The designed genus‐specific primers are proposed as rapid and cost‐effective tools to evaluate the most abundant bacterial genera in fish‐related or potentially other metagenomics samples.

## Graphical abstract

The design and testing of genus‐specific primers for rapid evaluation of the most abundant bacterial genera identified using amplicon‐based sequencing of the 16S rRNA gene in fish‐related samples.

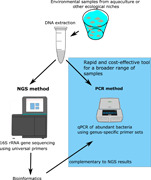

## 1 INTRODUCTION

The influence of symbiotic and pathogenic interactions of bacterial microbiota on terrestrial and aquatic vertebrates is of high interest (Sharpton, 2018). The advent of cost‐effective next‐generation sequencing (NGS) has opened up new avenues of culture‐free and high‐throughput analysis of the entire microbiota in any ecosystem and has significantly modified the understanding of their role in animal health and disease (Cao et al., 2017). As the 16 S rRNA gene is present in all bacteria, it is the most common reference gene for studies of bacterial phylogeny and taxonomy, and also for studies of the composition and the relative proportion of microorganisms in a given habitat (Janda & Abbott, 2007; Simon & Daniel, 2011). The structure of the 16S rRNA gene explains its versatility for metagenomics since universal primers can be designed in regions that are highly conserved across species and the intervening hypervariable regions can be used to assign operational taxonomic units (OTUs) to the genus taxon (Baker et al., 2003; Wang & Qian, 2009). Furthermore, the hypervariable regions offer the opportunity for the development of genus and even species targeted quantitative PCR (qPCR).

Aquatic organisms are exposed to a ubiquitous and abundant microbiota and the intensification of aquaculture has increased interest in characterizing the microbiota of fish. Initially traditional microbiological approaches based on in vitro culture were used, but more recently metagenomics approaches have been deployed (Martínez‐Porchas & Vargas‐Albores, 2017). Aquaculture differs from terrestrial farming systems as there is a much larger number of species exploited and this is coupled to a wide variety of environmental conditions (e.g., temperature and salinity) and geographical locations (Linhart et al., 2021). The variability of fish microbiota has been linked to geography, species, and environmental conditions and indicates that fish microbiomes have a degree of farm site‐specificity (Najafpour et al., 2021). Metagenomics studies targeting fish indicate that *Vibrio* and *Pseudomonas* are the dominant bacterial genera (reviewed by Egerton et al., 2018). However, the exclusive dependence on relative abundance data from NGS can lead to misinterpretation of microbial community structure (Jian et al., 2020). For this reason, it has been proposed that the use of genus‐specific primers can provide complementary, quantitative data, to corroborate NGS results and contribute to better understanding microbial diversity and population structure (Zhou et al., 2014). A general literature review of microbiome studies in fish reveals that genus‐specific primers for the most representative bacterial genera with high relative abundance are unavailable. Although genus‐specific primers exist for the detection of pathogen‐containing genera such as *Aeromonas*, *Vibrio*, *Edwardsiella*, and *Streptococcus*, their use has not been correlated with *16S rRNA* metagenomic profiles in fish (D. Zhang et al., 2014).

We previously generated *16S rRNA* metagenomics datasets for Gilthead seabream (*Sparus aurata*) and European seabass (*Dicentrarchus labrax*) eggs from several commercial production sites in Europe and identified the profile of the main bacterial genera (Najafpour et al., 2021). The objective of the present study was to develop a quick, cost‐effective, and practical approach for large‐scale screening of the core microbiome during aquaculture production cycles. We report the design of genus‐specific primers, exploiting the hypervariable characteristics of the 16S rRNA gene, for the dominant bacterial genera represented in our in‐house metagenomic 16S rRNA gene datasets from eggs, larvae, live feed, and tank water samples from seabream and seabass aquaculture sites in Europe (Najafpour et al., 2021). The efficiency and specificity of the genus‐specific primers were confirmed by qPCR and sequencing of the PCR amplicons, and coverage of each genus was validated by comparison of qPCR and metagenomics data (Najafpour et al., 2021).

## 2 EXPERIMENTAL PROCEDURES

### 2.1 Selection of target genera

**Figure 1:**
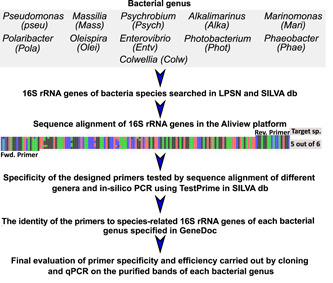
The workflow followed for the design of the bacterial *16S rRNA* genus‐specific primers for the most abundant genera in the microbiome of fish larvae, fish eggs, zooplankton, phytoplankton, and water. The most abundant genus detected in each data set was ranked using an in‐house 16S rRNA gene database. The egg microbiome profile obtained from 16S rRNA gene sequencing has previously been reported (Najafpour et al., 2021).

The summarized workflow for genus choice and primer design is presented in Figure 1. The most represented bacterial genera in seabream and seabass hatcheries in Europe were identified using in‐house metagenomic datasets of 16S rRNA gene sequences obtained from eggs, larvae, live feed, and hatchery water (sequenced by Lifesequencing S.L.‐ADM, Spain and Stab Vida, Lda, Portugal). The corresponding 16S rRNA gene sequences of target genera were obtained from the LPSN and Silva (SSU r138.1) databases (Parte et al., 2020; Quast et al., 2013).

### 2.2 Sequence alignment and primer design

Multiple sequence alignments of the retrieved 16S rRNA gene sequences from the LPSN and Silva databases were performed using the MUSCLE algorithm (Edgar, 2004) in the Aliview platform v 1.27 (Larsson, 2014). Genus‐specific forward (Fw) and reverse (Rv) primers were designed manually to obtain primers that amplified the maximum number of species in each of the target bacterial genera. The size of the amplicon was set at between 85 and 250 bp. Criteria used for PCR primer selection included the melting temperature (Tm), percentageof GC content, GC clamp, secondary structure, and the tendency to form primer‐dimers. Primers were analyzed and optimized using the oligonucleotide sequence calculator, OligoEvaluator™ (Sigma‐Aldrich, http://www.oligoevaluator.com) and OligoAnalyzer™ Tool (https://www.idtdna.com/calc/analyzer). In general, the threshold for primer selection included ΔG > −9 to minimize the likelihood of self‐dimers, hairpins, and heterodimers and 50%–55% GC content for both the Fw and Rv primers to favor specific annealing to the targeted templates (Table A1). Primers that did not comply with the selection criteria were rejected, apart from the primers for *Enterovibrio* for which it was not possible to meet all the criteria (Table A1).

To further confirm that the designed genus‐specific primer pairs would anneal to the maximal number of species in a given genus, in silico PCR simulations using TestPrime 1.0, available in the SILVA platform, was used (Klindworth et al., 2013). The primer pairs with the maximum specificity for each taxonomic group and with the best match to the selection criteria were selected and synthesized (Table A2, Specanalitica, Carcavelos, Portugal). On arrival, primers were resuspended in sterile, nuclease‐free water to prepare 100 µM stocks (Table A2).

### 2.3 Evaluation of primer specificity by PCR amplification

The performance of the genus‐specific primers was initially evaluated by running conventional PCR (Bio‐Rad, T100 Thermal Cycler). Genomic DNA was extracted from seabream and seabass eggs, whole larvae, and rotifers (feed) using a DNeasy Blood & Tissue Kit (Qiagen) as previously described (Najafpour et al., 2021). The PCR was carried out using 2 µl (approximately 40–80 ng) of genomic DNA, 2.5 µl of 10× Dream Taq Green Buffer containing 20 mM MgCl_2_ (Thermo Scientific), 0.5 µl of nucleoside triphosphate 10 µM stock, 1.25 µl of the Fw primer (10 µM), 1.25 µl of the Rv primer (10 µM), 0.2 µl of Dream Taq DNA polymerase (5 U/µl, Thermo Scientific), and 17.3 µl of sterile, nuclease‐free water to give a final reaction volume of 25 µl. The PCR thermocycle consisted of 1 cycle of 95°C for 3 min followed by 34 cycles of 95°C for 10 s, a gradient of melting temperatures tested for each primer pair (57–64°C) for 10 s and 72°C for 10 s and a final cycle at 72°C for 5 min. The genus‐specific primers were tested in PCR amplification of genomic DNA extracted from larval intestines, whole larvae, rotifers, and egg samples of seabream and seabass that had a high relative abundance of a given bacterial genus in *16S rRNA* metagenomics.

The 16S rRNA gene PCR reaction products were run on a 2% agarose gel in tris‐acetate‐EDTA buffer and the specific amplicons generated by each primer pair were purified using an Illustra GFX PCR DNA and Gel Band Purification Kit (GE Healthcare) following the manufacturer's instructions. The purified PCR products were ligated into the pGEM‐T Easy cloning vector (Promega) which permits colony selection based on white/blue color and resistance to the antibiotic ampicillin. The ligation reactions were performed overnight at 4°C in a 10 µl reaction mix containing 5 µl 2× Rapid Ligation buffer, 50 ng/µl pGEM®‐T Easy Vector, 1.5 U of T4 DNA Ligase (Promega), the optimal concentration of each of the purified PCR amplicons (between 19.8 and 80.5 ng) and sterile water, to give a final reaction volume of 10 µl. DH5α competent cells (*Escherichia coli*) were transformed with 5 µl of the ligation reaction, plated on lysogeny broth agar containing 75 µg/ml ampicillin, 0.5 mM isopropyli β‐d‐1‐thiogalactopyranoside, and 80 μg/ml X‐Gal (5‐bromo‐4‐chloro‐3‐indolyl β‐D‐Galactopyranoside), and incubated at 37°C overnight. Minipreps of plasmid DNA were prepared from white colonies and at least two colonies per genus were sequenced using the Sanger method.

The primer specificity and performance were evaluated by assigning taxonomy to each amplicon sequence using the SILVA 16S rRNA gene database as a reference and phylogenetic tree generation, based on the SILVA workflow (de novo including neighbors). For the phylogenetic tree, the sequences of the selected colonies were aligned using the Randomized Axelerated Maximum Likelihood (RAxML) tool with a generalised time reversible (GTR)model and Gamma rate model for likelihoods (Stamatakis, 2014).

### 2.4 Real‐time qPCR optimization and primer efficiency

After confirming primer specificity by amplicon sequencing, qPCR reactions were optimized using a Bio‐Rad CFX96 qPCR Instrument (Bio‐Rad Laboratories). The primer performance was initially evaluated using a gradient of melting temperatures (T_m_) in a reaction volume of 10 µl containing 200 nM of each primer, 2 µl of DNA (80 ng DNA/2 µl) for the samples, or 2 µl of serial dilutions (corresponding to 10^2^–10^7^ template copies in the reaction) for the standard curve, 5 µl of 2× Forget‐Me‐Not™ EvaGreen® qPCR Master Mix (Biotium) and 2.4 µl of sterile nuclease‐free water. Thermocycling conditions were 95°C for 2 min, followed by 40 cycles of 95°C for 5 s, the optimized melting temperature for each primer pair for 10 s (between 58 and 61°C) and 72°C for 10 s. A final melting curve was generated by increasing the temperature up to 95°C in increments of 0.5°C every 10 s to confirm single reaction products were obtained. Control reactions included substitution of genomic DNA by water to confirm the absence of contamination.

### 2.5 Bacterial genus quantification and correlation with *16S rRNA* microbiome profiling

To compare qPCR and 16S rRNA gene abundance estimates, Spearman correlations were used together with scatter plots generated using the R package ggplot2 v 3.3.5 (Wickham, 2016). *Colwellia*, *Oleispira*, *Phaeobacter*, *Pseudomonas*, and *Psychrobium* were quantified in nine egg samples (Najafpour et al., 2021, Table A3). *Massilia*, *Phaeobacter*, and *Pseudomonas* were quantified in seabream (*n* = 9; three larvae samples in the age range of 5–15 days posthatch [dph] and six samples in the age range of 43–58 dph) and seabass (*n* = 15; seven larvae samples in the age range of 5–7 dph and eight samples in the age range of 42–46 dph) larvae (Table A3). In the case of *Pseudomonas* where the correlation between 16S rRNA gene sequencing and copy number determined by qPCR was low, further in silico analysis was done to assess genus‐specific primer performance in comparison to the universal primers used for *16S rRNA* metagenomics studies. In silico PCR v 0.5.1 implemented in Ubuntu (20.04.2 LTS) and unique *Pseudomonas* 16S rRNA gene sequences from the Silva database with a minimum length of 900 bp were used.

## 3 RESULTS

### 3.1 Primer specificity

**Figure 2:**
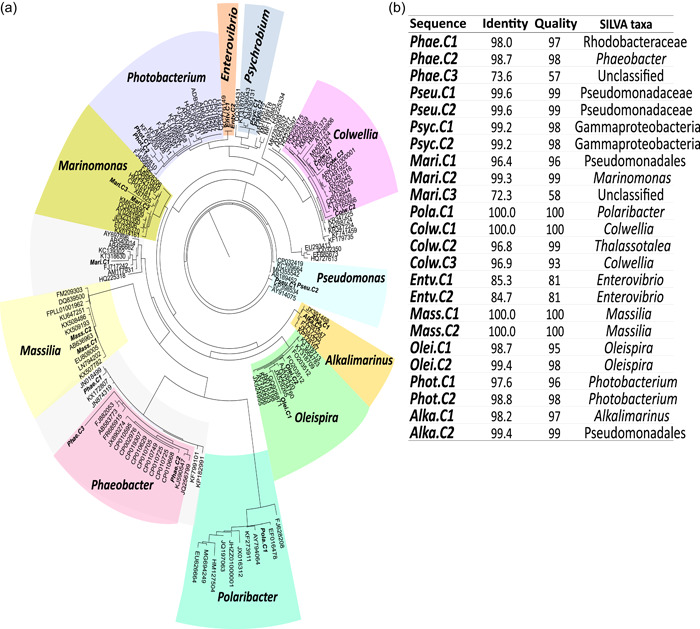
Analysis of the specificity of the 11 designed genus‐specific primer pairs by building a phylogenic tree using the 16S rRNA gene amplicon sequences and sequences retrieved from the SILVA database. (a) Phylogenetic clustering of the qPCR amplified and sequenced 16S rRNA genes, Phao.C1, Phao.C2, Phao.C3, Pseu.C1, Pseu.C2, Psyc.C1, Psyc.C2, Mari.C1, Mari.C2, Mari.C3, Pola.C1, Colw.C1, Colw.C2, Colw.C3, Entv.C1, Entv.C2, Mass.C1, Mass.C2, Olei.C1, Olei.C2, Phot.C1, Phot.C2, Alka.C1, Alka.C2 (NCBI accession number: OM685062–OM685080) and the 16S rRNA gene sequences retrieved from the SILVA database. The phylogeny was performed using the randomized axelerated maximum likelihood (RAxML) tool with a GTR model and Gamma rate model for likelihoods (Stamatakis, 2014). (b) Least‐common‐ancestor (LCA) sequence classification, which classified each amplicon based on the last level of classified taxa using the available sequences in the SILVA database. The alignment quality score and identity (%) for each amplicon alignment using the LCA method are presented. The number of neighbors per query sequence was set at 10. GTR, generalised time reversible.

**Figure A1:**
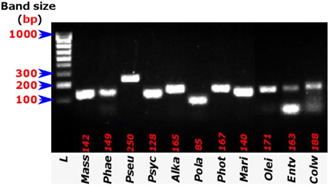
Analysis of PCR products (5 µl) on 2% agarose gel electrophoresis after staining with GreenSafe. The first lane (L) represents the DNA ladder and the size (base pair [bp]) of some markers is shown in red on the left‐hand side. Each lane contains the amplicon generated using the genus‐specific primer pairs (P) and sample type (S): P = *Massilia* (*Mass*), S = 49 days post hatch (dph) seabream larvae; P = *Phaeobacter* (*Phae*), S = 9 dph seabass larvae; P = *Pseudomonas* (*Pseu*), S = 46 dph seabass larvae; P = *Psychrobium* (*Psyc*), S = 39 dph seabream larvae; P = *Alkalimarinus* (*Alka*), S = intestine of seabream larvae, P = *Polaribacter* (*Pola*), S = intestine of seabream larvae; P = *Photobacterium* (*Phot*), S = intestine of seabream larvae; P = *Marinomonas* (*Mari*), S = rotifer; P = *Oleispira* (*Olei*), S = seabream eggs; P = *Enterovibrio* (*Entv*), S = intestine of seabream larvae; P = *Colwellia* (*Colw*), S = seabream eggs. The size of the PCR products amplified with each primer pair is presented in red (e.g., *Mass*, product size 142 bp). PCR, poymerase chain reaction.

In total, 11 bacterial genera were targeted based on their high relative abundance in fish‐related samples as determined by 16S rRNA gene sequencing (Table 1). The species for which the 16S rRNA genes were readily amplified by each primer pair are indicated in Table A4 and the supplementary figure at https://doi.org/10.5281/zenodo.6301068. Primer specificity was confirmed by the presence of a single band in agarose gel electrophoresis (Figure A1) and the sequence of the PCR amplicon. In the phylogenetic tree, most of the *16S rRNA* amplicon sequences generated clustered with the corresponding taxa in the SILVA 16S rRNA gene database, confirming the specificity of the genus‐specific primers (Figure 2). In the *Pseudomonas* clade, the sequence of the 16S rRNA gene amplicon from two samples clustered with several *Pseudomonas* species and included an uncultured *Pseudomonas* sp. (SILVA ID KP398534) and *Pseudomonas azotifigens* (AB189452; Figure 2a). In the *Massilia* clade, the sequence of the *16S rRNA* amplicons from two samples clustered with several *Massilia* species including *Massilia niastensis* (EU808005), and an uncultured *Massilia* sp. (AB636963). In the *Psychrobium* clade, the sequence of the 16S rRNA gene amplicons from two samples clustered with *Psychrobium* species, including an uncultured *Psychrobium* sp. (KT318702), *Psychrobium conchae* (AB930131; Figure 2a). In the *Phaeobacter* clade, the sequenced 16S rRNA gene amplicons clustered with *Phaeobacter* species, including *Phaeobacter inhibens* (CP010668), and *Sedimentitalea* sp. (JN018499; Figure 2a). In the *Colwellia* cluster, one of the 16S rRNA gene amplicons clustered with unidentified *Colwellia* spp. (L10950 and JX569143; Figure 2a) and the other amplicon clustered with *Thalassotalea* sp. (Figure 2b).

**Table 1:** Most abundant bacterial genera detected by *16S rRNA* metagenomics across different sample types

Bacterial genus	Maximum relative abundance of each bacterial genus in different samples (%)^a^						
	Fish egg	Fish larvae	Fish intestine	Rotifer	Artemia	Algae	Water
*Pseudomonas*	3.8	5.2	5.4	2.1	14.8	1.3	‐
*Massilia*	‐	36.1	1.2	‐	‐	2.2	89.6
*Psychrobium*	31.3	8.6	3.7	‐	‐	‐	33.4
*Phaeobacter*	‐	3.4	‐	‐	‐	‐	‐
*Marinomonas*	1.4	9	2.7	24.2	6.4	‐	14.6
*Polaribacter*	11.8	13.6	94.7	‐	‐	4.7	19.2
*Alkalimarinus*	1.9	3.2	29.4	‐	‐	‐	‐
*Enterovibrio*	‐	‐	94.4	‐	‐	‐	‐
*Photobacterium*	1.5	3.2	21.4	‐	‐	‐	40.8
*Oleispira*	6.6	2.2	1.7	‐	0.3	‐	8.3
*Colwellia*	9.5	4.8	1	‐	‐	‐	20.6

^a^ Identified in the 10 top bacterial genera in each sample type, (‐) signifies not detected within the most abundant bacterial genera of the specific sample type.

### 3.2 Primer efficiency and correlation between qPCR and 16S RNA gene abundance

The efficiency of bacterial genus‐specific primers was evaluated using qPCR with the optimized annealing temperature of each primer pair (Table 2). All the genus‐specific primers had an acceptable efficiency within the range of 92%–105.5%.

**Table 2:** List of bacterial genus‐specific primers designed and optimized for amplification of 16S rRNA genes in fish

Bacterial genus	Primer		Size (bp)	*T*_m_ (°C)	Eff. (%)	*R* ^2^
*Pseudomonas*	Pseu‐F	ACCGCATACGTCCTACGG	250	61	99.9	0.99
	Pseu‐R	CGAAGACCTTCTTCACACACG				
*Massilia*	Mass‐F	GCGTAGAGATGTGGAGGAAC	142	61	96.4	1.0
	Mass‐R	RACCCRACAACTAGTAGACATCG				
*Psychrobium*	Psyc‐F	GGAGGAAACTCTGATGCAGC	128	61	97.7	0.99
	Psyc‐R	GTCCTTCTTCTGCGAGTAACG				
*Phaeobacter*	Phae‐F	CACGTAGGCGGATCAGAAAG	149	61	97.6	0.99
	Phae‐R	GCCACTGGTGTTCCTCCG				
*Marinomonas*	Mari‐F	GAAGCACCGGCTAACTCTG	140	58.6	95.8	0.99
	Mari‐R	GTGCMATTCCAAGGTTGAG				
*Polaribacter*	Pola‐F	CTGGTTGACTTGAGTCATATGG	85	58	98.1	1.0
	Pola‐R	CGCAATCGGTATTCTGTG				
*Alkalimarinus*	Alka‐F	CGTAGGTGGTTTGTTAAGCGAG	165	61	95.9	1.0
	Alka‐R	GTCCAGTAAGTCGCCTTCG				
*Enterovibrio*	Entv‐F	GTGAGTAATGGCTGGGAACC	163	58	105.5	0.97
	Entv‐R	CTTGGTGAGCCATTACCTCAC				
*Photobacterium*	Phot‐F	TRGCCCAGGTGRGATTAG	167	58	92	1
	Phot‐R	GGCTGCATCAGGGTTTCC				
*Oleispira*	Olei‐F	CGGCTAATTTAGTGCCAG	171	58.2	95.2	1.0
	Olei‐R	CCACTAACCTCTCTCGTACTC				
*Colwellia*	Colw‐F	ATACGAGGGGTGCAAGCG	188	61	98.3	1.0
	Colw‐R	GATGTTCCTTCCAATCTCTACGC				

**Figure 3:**
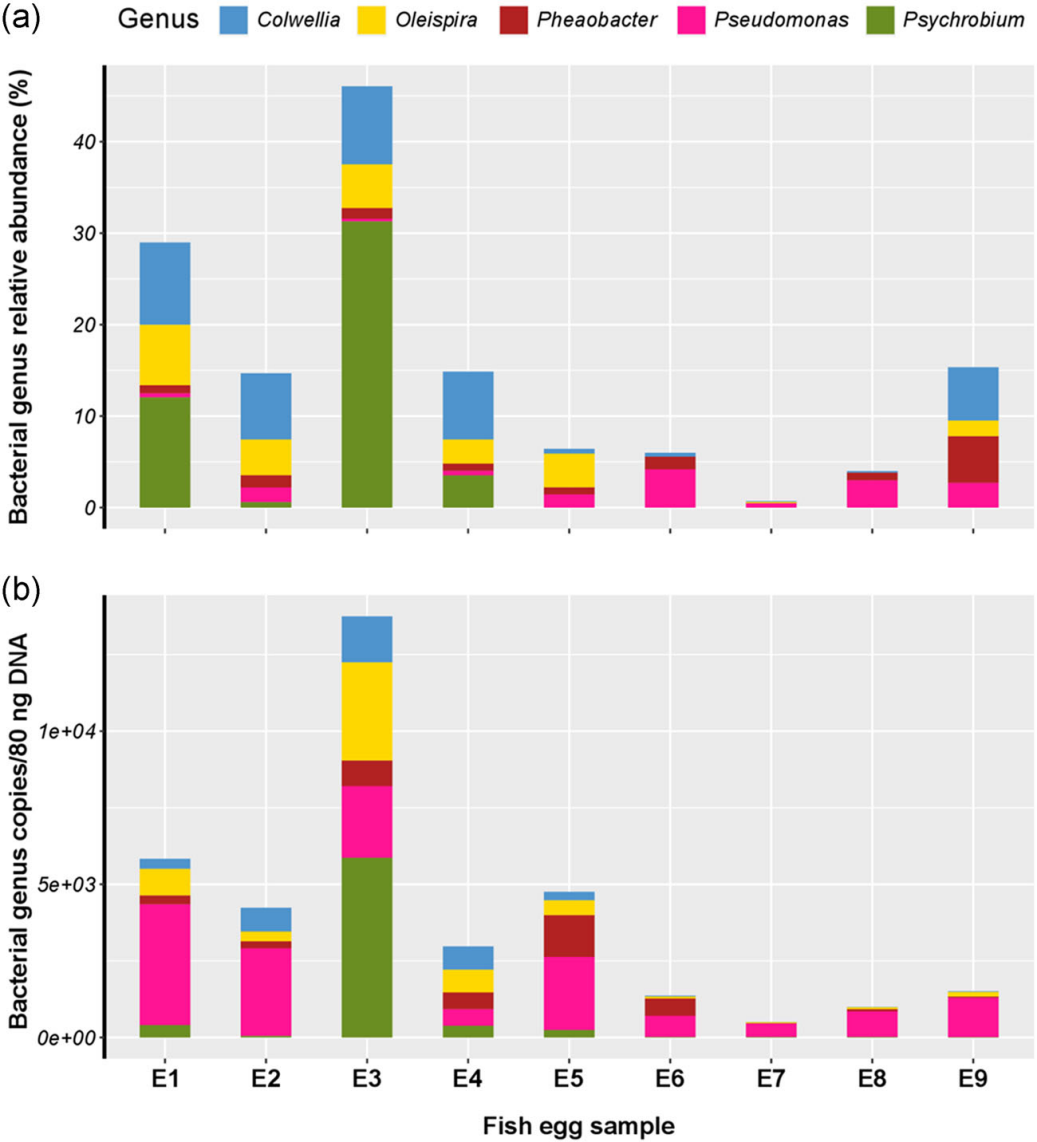
Analysis of the efficiency of five genus‐specific primer sets by comparing the relative abundance of the genus detected in seabream and seabass eggs using 16S rRNA gene sequencing and the copy number determined by qPCR. (a) The relative abundance (%) of five bacterial genera determined by 16S rRNA gene sequencing in nine egg samples from seabream (SA, *n* = 6) and seabass (DL, *n* = 3) from three different aquaculture sites (S1, S2, and S3). (b) Quantitative analysis of five bacterial genera in nine egg samples from seabream (*n* = 6) and seabass (*n* = 3) using qPCR with the designed genus‐specific primers. Full details of the experimental design, DNA extraction method, and the microbiome profile obtained using 16S rRNA gene sequencing of eggs samples are available in the Najafpour et al. (2021). The graphical plots were generated in the R package ggplot2 v 3.3.5. qPCR, quantitative polymerase chain.

**Figure 4:**
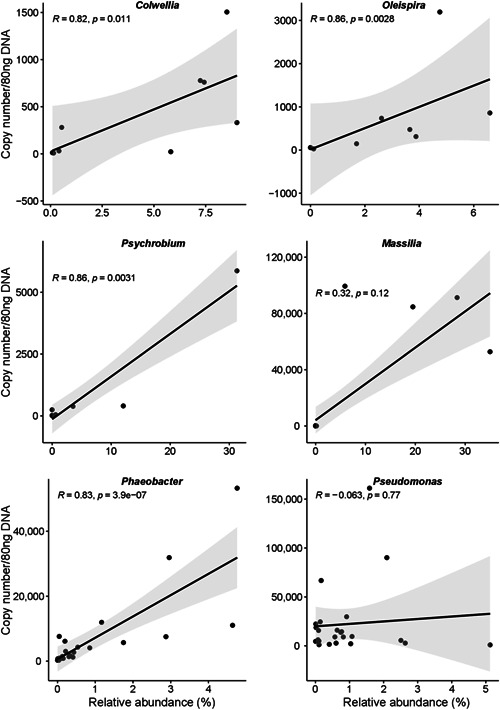
The correlation between genus‐specific copy number established by qPCR quantification and relative abundance of six bacterial genera determined by 16S rRNA gene sequencing of seabream and seabass egg samples. The scatter plots were generated using the “Spearman” method in an R environment. Two sets of samples and databases were used for the correlation analysis. (1) For the genera, *Colwellia*, *Oleispira*, and *Psychrobium*, the correlation analysis was established by comparing the relative abundance of the genera in nine egg samples of seabass (*n* = 3) and seabream (*n* = 6) obtained from 16S rRNA gene sequencing (Najafpour et al., 2021) and the copy number determined using genus‐specific primers and qPCR. (2) For the genera, *Massilia*, *Phaeobacter*, and *Pseudomonas*, the correlation analysis was established by comparing the relative abundance of the genera in 24 larval samples obtained from 16S rRNA gene sequencing (in‐house database) and copy number determined using genus‐specific primers and qPCR. The list of samples used in the analysis is available in Table A3. qPCR, quantitative polymerase chain.

The five most abundant bacterial genera in seabream and seabass eggs were quantified. In general, the relative abundance profiles of the 16S rRNA gene sequencing and qPCR amplification of different bacterial genera were matched with some exceptions (Figure 3). A significant positive correlation was obtained for the relative abundance (%) of bacterial genera detected by both methods (Figure 4). In egg samples (*n* = 9) highly significant positive correlations were found for *Colwellia* (*r* = 0.82), *Oleispira* (*r* = 0.86), and *Psychrobium* (*r* = 0.86, Figure 4). In seabream (*n* = 9) and seabass (*n* = 15) larvae, *Massilia* (*r* = 0.32) and *Phaeobacter* (*r* = 0.83) abundance also had significant positive correlations (Figure 4). For *Pseudomonas*, no correlation was found between the qPCR results for larvae (*r* = −0.063, *n* = 24) and egg (*r* = −0.22, *n* = 9) samples and the 16S rRNA gene sequencing (Figure 4). In silico PCR analysis comparing the *Pseudomonas*‐specific primers and the universal primers generally used for metagenomics (Klindworth et al., 2013) identified 27,734 and 26,780 unique sequences of the *Pseudomonas* 16S rRNA gene (total number in Silva = 60,869, filtered sequences number with the minimum size 900 bp = 41,607), respectively.

## 4 DISCUSSION

Genus‐specific primers for rapid and cost‐effective high‐throughput monitoring of core microbial genera of seabream and seabass aquaculture were successfully developed. The potential functional importance of the abundant bacterial genera selected makes further studies about their turnover during larval fish aquaculture important due to their high potential impact on production.

Several studies have focused on *Pseudomonas* spp. due to their widespread distribution and the presence of species that are human, animal, and plant pathogens (Palleroni, 2015). For example, *P. bactica* is a pathogen of marine fish, and primers targeting the *gyrB* gene (c390‐F1 and c390‐R1) and *rpoD* gene have been developed for rapid diagnosis (López et al., 2016). A multiplex PCR based on *oprI* and *oprL* genes was developed for the detection of *Pseudomonas* strains from a bacterial collection isolated from water (Matthijs et al., 2013). The qPCR comparisons, in the present study, of the abundance of five bacterial genera (*Colwellia*, *Oleispira*, *Pseudomonas*, *Psychrobium*, *Phaeobacter*) in the egg samples revealed a higher than expected copy number of the *Pseudomonas* genus relative to the results of the *16S rRNA* metagenomic sequencing. The *Pseudomonas* genus‐specific primer pair designed in the present study were of broad scope and had the potential to anneal to 226 out of the 254 16S rRNA gene sequences represented in the LPSN and SILVA databases. Insight into why a higher than expected copy number of *Pseudomonas* was detected by qPCR came from in silico PCR comparisons of the *Pseudomonas* genus‐specific and the 16S rRNA gene universal primers (Silva database, SSU r138.1) since it revealed the universal primers (Klindworth et al., 2013) had lower efficiency and annealed to fewer of the *Pseudomonas* sequences represented in the database. Furthermore, the relatively short amplification product generated by the *Pseudomonas* genus‐specific primers favors high qPCR efficiencies compared with previous primers designed to amplify a larger DNA sequence encompassing the ITS1 region (Locatelli et al., 2002). Bergmark et al. (2012) reported *Pseudomonas* and *Burkholderia* genus‐specific primers for their detection in soil and proposed the use of genus‐specific primers for both qPCR and 16S rRNA gene sequencing approaches to overcome issues related to the resolution of the 16S rRNA gene databases, although the exponential increase in available bacterial 16S rRNA gene sequences has now ameliorated this problem (Glöckner, 2019).

A high relative abundance of *Oleispira* and *Colwellia* genera was observed in our in‐house database of 16S rRNA gene sequences obtained for egg and water samples from aquaculture installations. The copy number of *Oleispira* and *Colwellia* in the same samples determined by qPCR using genus‐specific primers and their relative abundance using 16S rRNA gene sequencing gave a strong positive correlation. However, amplicon sequencing of products generated by the *Colwellia* primers yielded a *Colwellia‐*specific amplicon and another amplicon that matched the *Thalassotalea* genera of the Colwelliaceae family. The similarity between the sequence of the *Colwellia* genus‐specific primers and the 16S rRNA gene sequence of *Thalassotalea* (LPSN database) should be considered when using the primers. *Oleispira* and *Colwellia* are marine hydrocarbon‐degrading bacteria (Mason et al., 2014) and obligate hydrocarbonoclastic bacteria (OHCB) and are usually present in the environment in very low numbers (Golyshin et al., 2010). Pollution or the addition of hydrocarbons to water induces a rapid bloom of OHCB (Kasai et al., 2002) and indicates that variations in *Oleispira* and *Colwellia* abundance may be a useful indicator of excess hydrocarbons in aquaculture systems due, for example, to addition of lipid enriched feeds.

In seabream and seabass eggs, *Pseudophaeobacter* was relatively more abundant than *Phaeobacter*, which was not detected by 16S rRNA gene sequencing in eight out of nine egg samples (Najafpour et al., 2021). In contrast, *Phaeobacter* was more common in 16S rRNA gene sequences of seabream and seabass larvae, and the *Pseudophaeobacter* genus was not detected (in‐house database)*. Phaeobacter* spp. are common in marine organisms and the environment (Martens et al., 2006; Yoon et al., 2007; D. C. Zhang et al., 2008) and have been proposed as a probiotic against pathogenic *Vibrio* spp. in seabass and cod, *Gadus morhua* (D'Alvise et al., 2013; Grotkjær et al., 2016), although in juvenile squid they were associated with mortality (Won, 2009). The abundance‐dependent detection of *Phaeobacter* or *Pseudophaeobacter* in seabream and seabass samples highlights one of the challenges when designing primers for short amplicon targets of 16S rRNA genes that show high similarity across bacterial genera. The amplification of *Phaeobacter* or *Pseudophaeobacter* by the genus‐specific qPCR primers is unsurprising considering the recent reclassification of the species in *Leisingera‐Phaeobacter* to *Sedimentitalea* and *Pseudophaeobacter* (Breider et al., 2014). Therefore, it is proposed that the *Phaeobacter* genus‐specific primers designed in our study be designated group‐specific primers.

The primers for *Psychrobium* were highly specific and this genus was present in all analyzed samples of eggs, larvae, and environmental water and a positive correlation existed between the results of qPCR and 16S rRNA gene sequencing. The lowest number of species were detected for the *Psychrobium* genus compared with the other targeted genera and it was easier to design primers specific for this genus. The high specificity of the *Psychrobium* primers was assigned to the high variability of the *Psychrobium* 16S rRNA gene compared with other bacteria.

Six of the bacterial genera, *Massilia*, *Marinomonas*, *Polaribacter*, *Alkalimarinus, Enterovibrio*, and *Photobacterium*, for which genus‐specific primers were designed, had low relative abundance in the *16S rRNA* metagenome of eggs samples (Najafpour et al., 2021). Nonetheless, the identified genera were well represented in seabream and seabass larvae, rotifers, and tank water, and therefore genus‐specific primers were validated in qPCR. The designed genus‐specific primers revealed a high relative abundance of the *Massilia* genus in seabream and seabass larvae, *Marinomonas* in rotifer, and *Alkalimarinus*, *Polaribacter*, *Photobacterium, and Enterovibrio* in larval seabream intestine. These genera have previously been reported in a wide diversity of samples and experiments. For example, *Massilia* spp. are widespread in soil (Y. Q. Zhang et al., 2006), drinking water (Gallego et al., 2006), and plants (Ofek et al., 2012) and was a dominant genus in the gastrointestinal microbiota of the herbivorous grass carp, *Ctenopharyngodon idellus* (Li et al., 2014). *Marinomonas* species are abundant in seawater (Ivanova et al., 2005; Yoon et al., 2005) and were highly abundant in the microbial community of rotifer cultures prepared as a feed for fish larvae (Rombaut et al., 2001). *Polaribacter* species have been isolated from Antarctic soil and in biofilms on stones from the North Sea (Choo et al., 2020; Kim et al., 2013), and the genus was detected in the intestine of marine organisms and algae (Hyun et al., 2014; Nedashkovskaya et al., 2013; Wei et al., 2018). *Alkalimarinus sediminis* was isolated from marine sediment in Shandong Province, China (Zhao et al., 2015), and *Alkalimarinus* species were found in bone‐eating worms (*Osedax mediterranea*) from the Mediterranean Sea (Hewitt et al., 2020). Healthy and diseased *Dentex* and *Sparus aurata* (bony fishes) cultured in Spanish Mediterranean aquaculture contained *Enterovibrio coralii* strains and possibly *Enterovibrio nigricans* (Pascual et al., 2009). *Photobacterium* has been isolated from seawater, mussel, eggs of spiny lobster and fish intestine and the bioluminescence and pathogenicity of some species (e.g., *Photobacterium damselae*) has made studies of them a priority (Egerton et al., 2018; Labella et al., 2017; Osorio et al., 1999).

## 5 CONCLUSION

Genus‐specific primers were developed for *Pseudomonas*, *Massilia, Psychrobium, Phaeobacter, Marinomonas, Polaribacter*, *Alkalimarinus*, *Enterovibrio*, *Photobacterium*, *Oleispira*, and *Colwellia*. Most of the designed primers were highly efficient in qPCR and were genus‐specific as shown by amplicon sequencing and phylogeny. Difficulty was encountered in the design of genus‐specific primers for *Phaeobacter* and *Pseudophaeobacter* since the short amplicons of the 16S rRNA gene encompassed regions with 93%–98% identity and so these primers were designated group‐specific. The genus‐specific primers designed in this study will be useful for rapid evaluation and quantification by qPCR of target bacterial genera in fish‐related samples and potentially other metagenomic samples since the genus targeted are ubiquitous in a diversity of environments. An interesting observation was that the 16S rRNA gene universal primers used to profile microbiomes do not cover all identified species of some bacterial genera such as *Pseudomonas* and this has the potential to create bias in metagenomics studies and reinforces the value of complimentary qPCR studies. Overall, the designed genus‐specific primers provided a rapid and cost‐effective evaluation of abundant bacterial genera in samples and can therefore contribute to understanding the modulation of abundant microbiota in complex microbial communities with a potentially high impact on host biology.

## CONFLICTS OF INTEREST

None declared.

## ETHICS STATEMENT

None required.

## AUTHOR CONTRIBUTIONS

**Babak Najafpour**: Formal analysis‐lead, investigation‐lead, methodology‐lead, Writing–original draft‐lead; **Patricia Pinto**: Formal analysis‐supporting, methodology‐supporting, writing–review & editing‐dupporting**; Adelino V. M. Canario**: Methodology‐supporting, project administration‐supporting, resources‐supporting, supervision‐supporting, writing–review & editing‐supporting**; Deborah M. Power**: Conceptualization‐lead, formal analysis‐supporting, funding acquisition‐lead, investigation‐lead, methodology‐supporting, project administration‐lead, resources‐equal, supervision‐lead, writing–original draft‐supporting, writing–review & editing‐lead.

## Data Availability

All data are provided in full in this paper apart from amplicon sequences of 16 S rRNA genes which are available at www.ncbi.nlm.nih.gov under accession numbers OM685062‐OM685080. The supplemental figure is available in Zenodo at https://doi.org/10.5281/zenodo.6301068 (The alignment and identity of 16 S rRNA genes and genus‐specific primer sequences for different bacterial species of each target bacterial genus).

## 1

**Table A1:** Evaluation of primer GC content, dimer and secondary structure formation, and length using the OligoAnalyzer™ Tool

Bacterial genus primer pair		GC (%)	Self‐dimer (ΔG kcal. mole^−1^)	Hetero‐dimer (ΔG kcal. mole^−1^)	Hairpin (ΔG kcal. mole^−1^)	Length (bp)
*Pseudomonas*	Fw	61.1	−6.68, −0.96	−4.95, −1.34	−2.18, −1.97	18
	Rv	52.4	−6.69, −1.57		−3.24, −3.13	21
*Massilia*	Fw	55	−3.61, −0.96	−6.35, −1.34	0.39,1.24	20
	Rv	47.8	−6.84, −0.96		0.01, 0.28	23
*Psychrobium*	Fw	55	−7.05, −1.47	−6.69, −1.34	−2.86, −1.87	20
	Rv	52.4	−3.61, −0.96		−1.17	21
*Phaeobacter*	Fw	52.4	−3.61, −0.96	−3.61, −1.34	−1.17	21
	Rv	66.7	−5.02, −1.34		−0.97, −0.41	18
*Marinomonas*	Fw	57.9	−9.75, −0.96	−6.44, −1.34	−2.04	19
	Rv	50	−7.6, −1.57		−0.73	19
*Polaribacter*	Fw	45.5	−7.82, −1.34	−3.9, −1.34	−2.42	22
	Rv	50	−3.61, −1.47		−0.27, 0.54	18
*Alkalimarinus*	Fw	50	−4.85, −0.96	−8.33, −0.96	−0.11, 0.18	22
	Rv	57.9	−3.61, −0.96		−0.52, −0.4	19
*Enterovibrio*	Fw	55	−3.14, −0.96	−15.48, −1.34	−1.25	20
	Rv	52.4	−6.47, −0.96		−3.04	21
*Photobacterium*	Fw	55.6	−8.54, −0.96	−8.54, −1.46	0.9, 1.57	18
	Rv	61.1	−7.05, −1.47		−0.87, −0.09	18
*Oleispira*	Fw	61.1	−7.05, −1.47	−6.36, −1.34	−0.87, −0.09	18
	Rv	52.4	−3.65, −0.96		1.37, 1.41	21
*Colwellia*	Fw	61.1	−7.05, −0.96	−6.75, −0.96	−0.55, 0.15	18
	Rv	47.8	−3.61, −0.96		0.49, 1.46	23

*Note*: ΔG = free energy required to break the structure, OligoAnalyzer™ Tool (https://www.idtdna.com/calc/analyzer).

**Table A2:** Details of the genus‐specific primers and their preparation for qPCR

Primer target		Amount received (nmol)	Primer Melting temp. (°C)	Optical density (260 nm)	Sterile water^a^ (µl)
*Pseudomonas*	Fw	9.7	57.2	2	97
	Rv	10.2	65.1	2	102
*Massilia*	Fw	9.5	55.4	2	95
	Rv	7.8	55.5	2	78
*Psychrobium*	Fw	10.1	56.2	2	101
	Rv	10.5	55.6	2	105
*Phaeobacter*	Fw	9.7	55.9	2	97
	Rv	12.8	59.7	2	128
*Marinomonas*	Fw	11.1	56.3	2	111
	Rv	9.9	52.8	2	99
*Polaribacter*	Fw	9.5	52.8	2	95
	Rv	11.9	50.8	2	119
*Alkalimarinus*	Fw	9.2	56.6	2	92
	Rv	11.3	55.9	2	113
*Enterovibrio*	Fw	10	56.0	2	100
	Rv	10.5	55.9	2	105
*Photobacterium*	Fw	9.7	53.7	2	97
	Rv	12.2	57.2	2	122
*Oleispira*	Fw	11.6	50.6	2	116
	Rv	11	54.0	2	110
*Colwellia*	Fw	10.8	58.4	2	108
	Rv	9.7	55.5	2	97

Abbreviations: Fw, forward primer; qPCR, quantitative polymerase chain reaction; Rv, reverse primer.

^a^ Microliter of sterile nuclease‐free water added to the lyophilized primers to give a 100 µM stock.

**Table A3:** List of samples used to correlate qPCR and 16S rRNA gene abundance estimates

Sample^a^	Sample type	Site	Species	Sample code	Sequenced reads^b^
E1	Egg	Site 1	Seabream	S1.SA.E.AD3	370,906
E2	Egg	Site 1	Seabream	S1.SA.E.AD4	513,514
E3	Egg	Site 1	Seabream	S1.SA.E.BD3	409,804
E4	Egg	Site 1	Seabream	S1.SA.E.BD4	312,384
E5	Egg	Site 2	Seabream	S2.SA.E.AD1	417,468
E6	Egg	Site 2	Seabream	S2.SA.E.BD1	587,762
E7	Egg	Site 3	Seabass	S3.DL.E.BD2	628,184
E8	Egg	Site 3	seabass	S3.SA.E.AD2	616,148
E9	Egg	Site 3	Seabass	S3.SA.E.BD2	522,126
L1–L3	Larvae, 5–15 dph	F, G, I	Seabream	SA.1.JM	150,859^b^
L4–L10	Larvae, 5–7 dph	A, F, H, E, D, G	Seabass	DL.1.JM	72,736^b^
L11–L16	Larvae, 39–58 dph	A, B, C, F, G, H	Seabream	SA.3.JM	105,275^b^
L17–24	Larvae, 42–46 dph	A, C, D, E, F, G, H	Seabass	DL.3.JM	55,062^b^

Abbreviations: dph, days posthatch; qPCR, quantitative polymerase chain reaction.

^a^ Nine egg samples (E1–E9) used for qPCR reactions of five bacterial genera (*Pseudomonas*, *Oleispira*, *Colwellia*, *Psychrobium*, and *Phaeobacter*), and the details of their 16S rRNA gene sequencing profiles are available in Najafpour et al. (2021). “Factors driving bacterial microbiota of eggs from commercial hatcheries of European seabass and gilthead seabream” and the metagenomics raw data were deposited at NCBI SRA (sequence read archive) under project number PRJNA727018. Twenty‐four larvae samples (L1–L24) and their corresponding qPCR and 16S rRNA gene sequencing data (available as in‐house datasets) were also used to correlate qPCR and 16S rRNA gene abundance estimates of three genera (*Massilia*, *Phaeobacter*, and *Pseudomonas*).

^b^ For larvae samples, the average number of the sequenced reads is presented for each age range.

**Table A4:** The numbers of potentially amplified and unamplified bacterial species with each of the designed genus‐specific primer set

Primer	Total number of species^a^	Number of amplified species	Unamplified species
*Pseu*	254	226	*P. luteola* (AB681955), *P. kirkiae* (MK159379), *P. ianjinensis* (MF083697), *P. asuensis* (AJ871471), *P. caeni* (EU620679), *P. oryzihabitans* (AB681726), *P. zeshuii* (JN411093), *P. duriflava* (EU046271), *P. litoralis* (FN908483), *P. psychrotolerans* (AJ575816), *P. rhizoryzae* (MK759856).
*Mass*	49	48	*M. brevitalea* (EF546777)
*Psyc*	1	1	‐
*Phae*	6	5	*P. italicus* (AM904562)
*Mari*	33	31	*M. ushuaiensis* (AJ627909), *M. algicida* (KR003453)
*Pola*	26	20	*P. marinivivus* (KM017972), *P. huanghezhanensis* (MH791326), *P. aestuariivivens* (MH791326), *P. porphyrae* (AB695286), *P. lacunae* (KJ728849), *P. pacificus* (KU315476)
*Alka*	1	1	‐
*Photo*	38	35	*P. galatheae* (FJ457476), *P. salinisoli* (KP054474), *P. halotolerans* (AY551089)
*Olei*	2	2	‐
*Colw*	21	19	*C. demingiae* (U85845), *C. hornerae* (JN175346)
*Entv*	6	5	*E. pacificus* (JN118548)

^a^ 16S rRNA genes of bacterial species present in the LPSN db (linked to the Silva db); a full list of the sequences used in the alignments is presented in the supplemental figure at https://doi.org/10.5281/zenodo.6301068
